# Intercalation of pyrazolone-based oxalamide metal complexes into Na-montmorillonite for catalytic liquid-phase oxidation of phenol using H_2_O_2_

**DOI:** 10.55730/1300-0527.3630

**Published:** 2023-10-10

**Authors:** Serhat UZAN, Eyüp BAŞARAN

**Affiliations:** 1 Food Analysis Application and Research Center (BÜGAM), Batman University, Batman, Turkiye; 2 Department of Chemistry and Chemical Processing Technologies, Vocational School of Technical Sciences, Batman University, Batman, Turkiye

**Keywords:** Intercalation, oxidation of phenol, oxalamide, Na-montmorillonite, catalyst

## Abstract

The intercalation of pyrazolone-based oxalamide metal complexes into Na-montmorillonite (Na-MMT) for catalytic liquid-phase oxidation of phenol using H_2_O_2_ was undertaken by a flexible ligand method using metal ions including Mn(II), Zn(II), and Sn(II). First, the metal ions were exchanged with the sodium ions of Na-MMT, and then these metal ions were complexed with a new pyrazolone-based oxalamide ligand. The intercalated metal complexes were characterized by Fourier transform infrared spectroscopy, X-ray diffraction, scanning electron microscopy, energy-dispersive X-ray spectroscopy, elemental analysis, and thermogravimetric analysis. Phenol was successfully oxidized by heterogeneous catalysts based on Mn(II), Zn(II), and Sn(II) pyrazolone-based oxalamide complexes intercalated into Na-MMT. These heterogeneous catalysts catalyze the liquid-phase oxidation of phenol using H_2_O_2_ to catechol as the major product and hydroquinone and benzoquinone as the minor products. The Mn(II) and Zn(II) complexes intercalated into Na-MMT showed better activity than the Sn(II) complex intercalated into Na-MMT and the reaction without the catalyst. It has been shown that some metal ion complexes intercalated into Na-MMT are active catalysts for liquid-phase oxidation of phenol with hydrogen peroxide.

## 1. Introduction

Chemical industry applications of the hydroxylation of phenol to catechol and hydroquinone include the production of polymerization inhibitors, petrochemicals, oil refineries, textiles, paints, foods, photographic chemicals, antioxidants, pesticides, flavoring agents, and medicine [1–5]. Catechol is commonly utilized in industry as an important intermediate in the manufacturing of pesticides and drugs, and it is also utilized in the manufacturing of insecticides such as carbofuran and propoxur [6]. The aromatic C-H bond is so energetically stable that direct catalytic hydroxylation of inert C-H bonds in hydrocarbons under mild conditions is one of the most difficult to perform in synthetic chemistry, academic research, or the chemical industry [7,8].

Selective oxidation is of significant industrial importance owing to its application in the synthesis of chemical intermediates necessary for the production of high-tonnage commodities, valuable fine chemicals, and essential ingredients. Despite its important role, however, this process is inefficient [9]. The utilization of solid catalysts in the selective oxidation of phenols, particularly under near-ambient conditions, and the utilization of clean oxidants such as O_2_ or H_2_O_2_, is an increasingly significant area of research [10]. Recently, transition metals that directly catalyze the hydroxylation of inert C-H bonds in hydrocarbons have emerged as a strong means for the regioselective synthesis of phenols [11].

The quest for cost-effective and ecofriendly catalysts has led to a proclivity towards heterogeneous catalysts in research endeavors, owing to their superiority over their homogeneous counterparts, particularly in terms of facile extraction from reaction mixtures, specificity, and reusability [12]. Heterogenized materials consisting of transition metal complexes exhibiting catalytic activity in inorganic supports exhibit chemical selectivity similar to that of their original homogeneous counterparts [10]. However, these materials demonstrate greater chemical stability and confer some of the benefits commonly associated with heterogeneous catalysts, such as facile separation from reaction media, reusability, and thermal stability [10,13]. Thus, the use of these materials has the potential to promote greater environmental sustainability [13,14].

Clays are widely available in nature as low-cost chemical substances. Clays are ambidextrous materials that catalyze different chemical reactions, both in their natural form and in many modified forms [15]. Layered clay minerals serve as structures for the immobilization of metal complexes [16]. Layered silicates have rich intercalation ability and high thermal and chemical stability, are abundant in nature, and are inexpensive. Therefore, layered silicates can potentially find widespread applications as adsorbents, catalysts, and catalytic scaffolds [17]. Additionally, layered silicates exhibit interlayer reactions such as cation exchange and grafting because the interlayer surface is abundantly covered with silanol groups (SiO- or SiOH). For this reason, they have been investigated as cation exchangers, and their high cation-exchange capacities have been demonstrated compared to clay minerals and zeolites [18]. The clay mineral montmorillonite (MMT), a member of the dioctahedral smectite group, is the most important smectite used in catalytic applications [19,20]. The structure is composed of a stacked arrangement of TOT layers, wherein an alumina octahedral sheet (O) is situated between two silica tetrahedral sheets (T). It is commonly observed that the isomorphic substitution of Al^3+^ by Mg^2+^ occurs within the octahedral sheet [21]. Owing to the replacement of clay in the octahedral layer by cations of lower valence, a superfluous negative charge is manifested on the surface of the packet. The negative electrical charge present in the interpacket region is counterbalanced by cations that can be exchanged, namely Na^+^, K^+^, Ca^2+^, and Mg^2+^ [22, 23]. There are two distinct natural variations in MMT: sodium MMT, which exhibits a remarkable capacity for swelling in water, and calcium MMT, which displays comparatively modest swelling capacity [22]. MMT can be easily replaced by various metallic ions, including Al^3+^, Sn^2+^, and Ti^4+^. Owing to its extensive cation-exchange capacity, MMT permits facile immobilization of various cations comprising individual metallic ions and cationic metallic complexes through ion-exchange approaches [24]. The catalytic performance of metal ion-exchanged MMT was determined by the presence of ions. Additionally, the expandable interlayer space allows for the inclusion of organic molecules, thus enabling variations in interlayer distances, hydrophobicity, adsorption, and catalytic applications [25].

In this study, Mn and Zn, which are first-row transition metals that are most abundant on the surface of the earth and offer low costs, little or no toxicity, and unique catalytic properties, were selected for intercalation into the interlayers of Na-MMT. At the same time, the catalytic effect of nontransition metal Sn intercalated to Na-MMT was investigated and compared with that of the transition metals used in this study. Complexes of Mn^2+^, Zn^2+^, and Sn^2+^ ions with a new ligand previously synthesized by us were intercalated into Na-MMT, which has a large cation-exchange capacity. Liquid-phase oxidation of phenol was carried out with the oxidizing agent H_2_O_2_ in the presence of metal complexes intercalated into Na-MMT as catalysts. The conversion of phenol to catechol (CAT), benzoquinone (BQ), and hydroquinone (HQ), the main oxidation products, was kinetically investigated.

## 2. Experimental

### 2.1. Materials

Analytical reagent-grade metal chlorides (ZnCl_2_, MnCl_2_.4H_2_O, and SnCl_2_.2H_2_O) were procured from Sigma-Aldrich (St. Louis, MO, USA). Sodium montmorillonite (Na-MMT), a hydrated aluminum silicate with sodium as the predominant exchangeable cation (Southern Clay Products, Inc., Gonzales, TX, USA), is a powder with a particle size of 10 to 20 μm. The specific gravity of the Na-MMT was 2.86 and the molecular weight was 540.46 g/mol; the pH of the 3% suspension of Na-MMT was 8, and its cation-exchange capacity as reported by the supplier was 92 mEq/100 g clay. Hydrogen peroxide (50 wt.% in H_2_O) was obtained from Sigma-Aldrich. Methanol and chloroform were also purchased from Sigma-Aldrich. The pyrazolone-based oxalamide ligand, which we named *N*,*N*′-di(antipyrin-4-yl)oxalamide (DAO), was prepared as previously published [26].

### 2.2. Instrumentation

Fourier transform infrared (FTIR) spectra were measured in the range of 4000 to 400 cm^−1^ using a Shimadzu-IR Tracer 100 FTIR Spectrometer (Shimadzu Corp., Kyoto, Japan). Powder X-ray diffraction (XRD) patterns were obtained using a Rigaku Miniflex II diffractometer (Rigaku Corp., Tokyo, Japan) with monochromatic Cu-Kα radiation. A DTG-60 device (Shimadzu Corp.) was used for thermogravimetric and differential thermal analyses. Elemental analysis was performed using a Thermo Scientific FLASH 2000 series instrument (Thermo Fisher Scientific, Waltham, MA, USA). A GC-FID Shimadzu GC-2030 instrument was fitted with a flame ionization detector. Scanning electron microscopy (SEM) and energy-dispersive X-ray spectroscopy (EDX) analyses were performed using an FEI Quanta 250 FEG field scanning microscope (FEI, Hillsboro, OR, USA).

### 2.3. Preparation of metal [Cu(II), Ni(II), and Sn (II)]-exchanged montmorillonite

One gram of Na-MMT was suspended in 100 mL of 0.1 M metal chloride [chlorides of Mn(II), Zn(II), and Sn(II)] solution. Additionally, a Sn(II) chloride solution was prepared in 0.05 N HCl solution. The resulting suspension was magnetically stirred for 24 h. The solid was filtered under vacuum, and the filtrate was washed with distilled water until free metal ions were removed from the surface of the MMT and then dried in an oven at 110 °C for 24 h.

### 2.4. Preparation of metal-oxalamide complexes intercalated into montmorillonite

**Figure 1:**
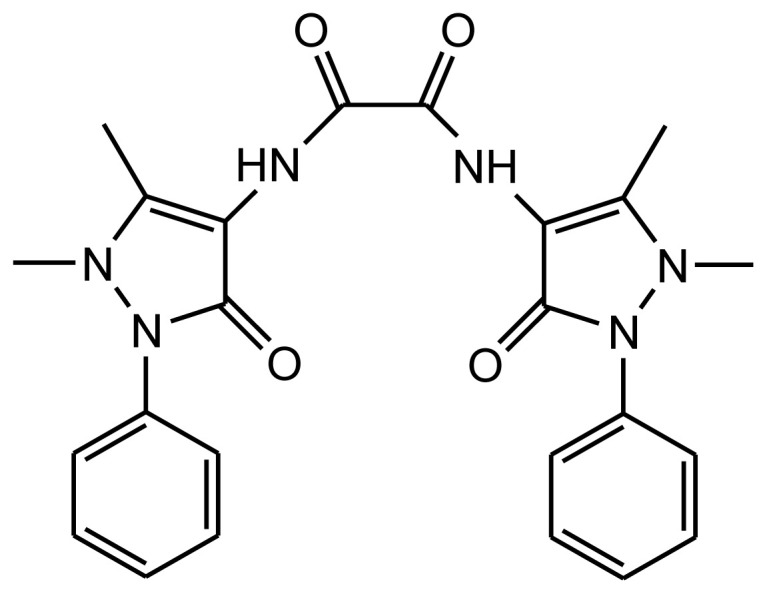
Structure of *N*,*N*′-di(antipyrine-4-yl) oxalamide (DAO).

One gram of metal ion-exchanged MMT was suspended in 50 mL of a methanol:chloroform (1:1, v/v) solvent mixture. As shown in Figure 1, 0.27 g of ligand (DAO, 0.575 mmol) was added to this suspension at a metal:ligand ratio (1.0:1.25, mmol/mmol) corresponding to the cation-exchange capacity of the clay. The mixture was refluxed for 9 h with stirring. The material was washed several times by centrifugation with a methanol:chloroform (1:1, v/v) solvent mixture to remove free ligand or complex on the surface of the MMT. The solid was dried at 60 °C and ground.

### 2.5. Catalytic oxidation of phenol

The phenol oxidation reaction was carried out in the liquid phase in the presence of a catalyst by refluxing with the addition of H_2_O_2_. The liquid organic products were quantified by comparison with standards using gas chromatography. In a typical reaction procedure, a mixture of 0.005 mol (0.470 g) phenol (dissolved in 2 mL of organic solvent) and an aqueous solution of 50% H_2_O_2_ (3.4 g, 0.05 mol) was added to a 50-mL one-necked flask equipped with a condenser. The reaction mixture was then heated to 80 °C under continuous stirring. Subsequently, 0.020 g of the catalyst was added to the reaction mixture. After the reaction was complete, the catalyst was centrifuged and analyzed by dilution with acetonitrile. A GC-FID Shimadzu GC-2030 instrument was fitted with a flame ionization detector with an Rxi-5ms column of length 30 m and 0.25 ID using He as the carrier gas. The temperature program was as follows: the temperature of the column was maintained at 40 °C for 5 min, then raised to 270 °C (10 °C/min) and then maintained at 270 °C for 5 min. Calibration curves were built in advance using the areas below each peak for CAT, BQ, and HQ. The temperatures of both the injector and detector were maintained at 270 °C.

## 3. Results and discussion

### 3.1. Characterization of heterogeneous catalysts

#### 3.1.1. Fourier transform infrared spectroscopy

**Figure 2:**
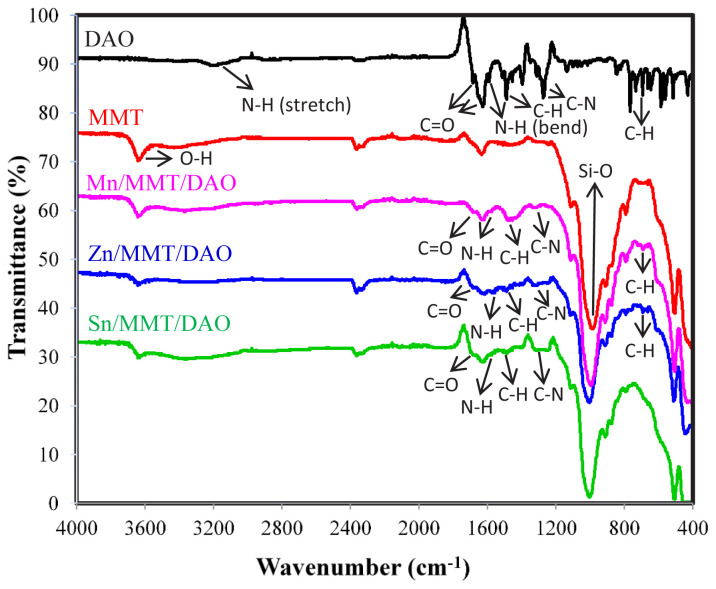
FTIR spectra of ligand: DAO, crude clay: Na-MMT, metal-oxalamide complexes intercalated into MMT: Mn/MMT/DAO, Zn/MMT/DAO, Sn/MMT/DAO.

The FTIR spectra of the metal-oxalamide complexes intercalated into MMT are presented in Figure 2. Characteristic absorption peaks of the metal-oxalamide complexes were observed in the FTIR spectra of the metal-oxalamide complexes intercalated into MMT. In the free oxalamide ligand, the amide carbonyl C=O stretching frequency was observed at 1685 cm^−1^ [26], while in the Mn/MMT/DAO, Zn/MMT/DAO, and Sn/MMT/DAO, which are metal-oxalamide complexes intercalated into the MMT amide, carbonyl C=O stretching frequencies were observed at 1683, 1685, and 1685 cm^−1^, respectively. The band at 1593 cm^−1^ corresponding to the amide N-H bending frequency of the free DAO ligand shifted to a lower frequency in the case of the metal-oxalamide complexes intercalated into MMT for Mn/MMT/DAO, Zn/MMT/DAO, and Sn/MMT/DAO at 1585, 1577, and 1577 cm^−1^, respectively, because of the coordination of the amide nitrogen of DAO with metal ions. The C-N stretching band in the frequency range of 1371–1226 cm^−1^ in the free ligand appeared as broad bands in the frequency range 1369–1226 cm^−1^ in all three metal-oxalamide complexes intercalated into MMT. The peak at 700 cm^−1^, corresponding to the C-H bending frequency of the free DAO ligand, was observed at 700 and 698 cm^−1^ in Mn/MMT/DAO and Zn/MMT/DAO, respectively.

#### 3.1.2. Thermal studies

**Figure 3:**
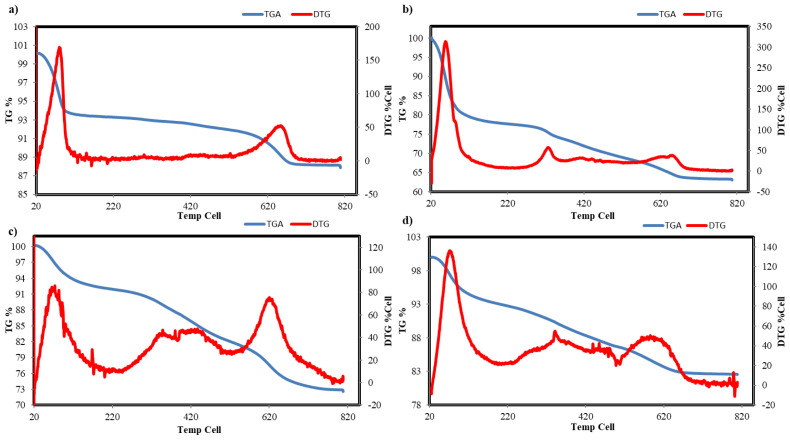
TG/DTG curves of a) Na-MMT, b) Mn/MMT/DAO, c) Zn/MMT/DAO, and d) Sn/MMT/DAO.

The thermograms of Na-MMT and the metal-oxalamide complexes intercalated into MMT in Figures 3a–3d along with the group loss and the percentage weight loss due to temperature increase at different steps are presented in Table 1. While the thermal decomposition of Na-MMT occurs in two major steps, that of the metal-oxalamide complexes intercalated into MMT occurs in three major steps. In the first step, a weight loss of 6.30%–21.59% occurs between 20 and 160 °C owing to the presence of free water. The second step involves weight loss of 6.21%–9.09% between 184 and 484 °C due to the decomposition of the chelating ligand in all three metal-oxalamide complexes intercalated into MMT. This second step, which occurs due to the degradation of the chelating ligand, which is further evidence for the complexing of the metal-MMT, is absent in the thermogram of MMT. The third step, which entails weight loss of 4.03%–10.44% between 480 and 800 °C, is associated with the loss of the structural hydroxyl group.

#### 3.1.3. EDX/SEM and elemental analysis

**Figure 4:**
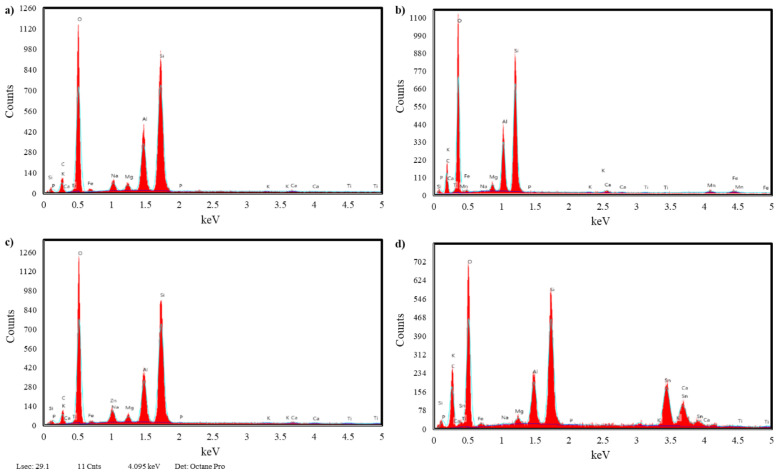
EDX spectra of a) Na-MMT, b) Mn/MMT, c) Zn/MMT, and d) Sn/MMT.

**Figure. 5:**
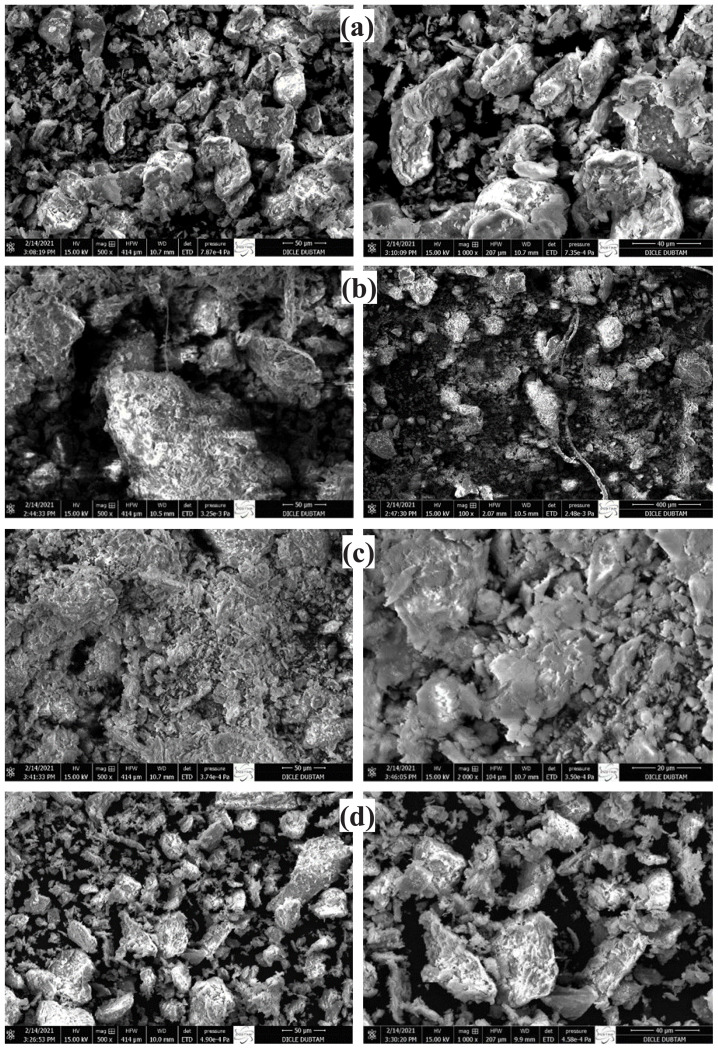
SEM images of a) Na-MMT, b) Mn/MMT, c) Zn/MMT, and d) Sn/MMT.

The EDX analysis results of the original Na-montmorillonite clay and the clay after it was exchanged with Mn(II), Zn(II), and Sn(II) ions are summarized in Table 2. The ten elements of sodium (Na), potassium (K), magnesium (Mg), calcium (Ca), oxygen (O), phosphorus (P), silicon (Si), titanium (Ti), aluminum (Al), and iron (Fe) in the EDX spectra (Figures 4a–4d) represent the components of the MMT. The EDX results showed that Na-MMT contains all elements except Mn, Zn, and Sn. The weight percentages of Mn, Zn, and Sn were 1.70% in Mn/MMT, 7.91% in Zn/MMT, and 28.69% in Sn/MMT, respectively. In addition, the SEM results of the materials showed the presence of well-defined MMT crystals without any shadow of metal ions or complexes on their outer surfaces (Figures 5a–5d).

As shown in Table 3, the elemental analysis results showed that the amount of nitrogen and carbon increased after the preparation of the metal-oxalamide complexes intercalated into MMT. As the amount of ligand complexed in MMT increased, the amount of nitrogen and carbon increased; therefore, the amount of ligand increased in the following order: Zn/MMT/DAO > Mn/MMT/DAO > Sn/MMT/DAO.

#### 3.1.4. XRD diffraction study

**Figure 6:**
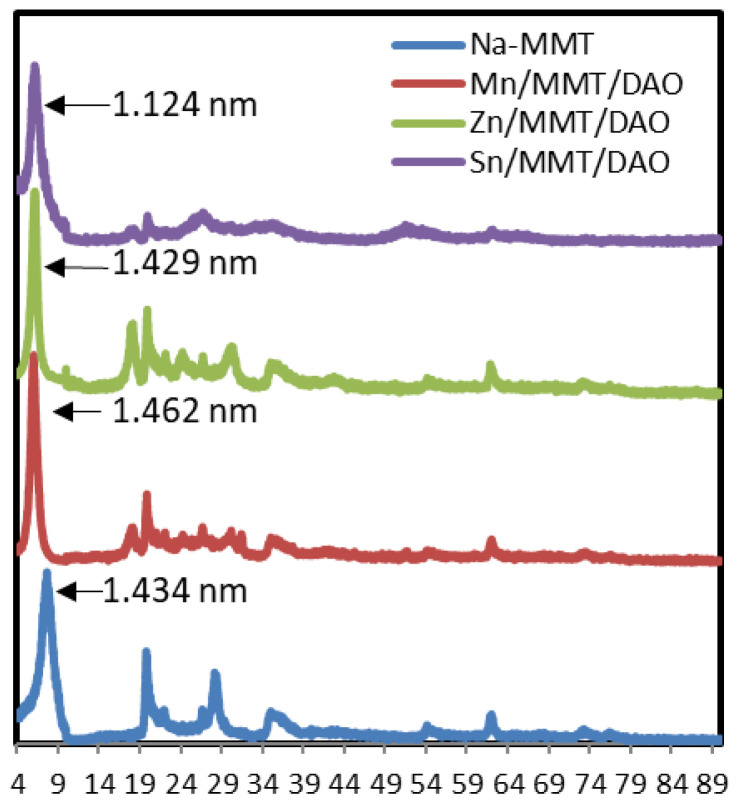
X-ray diffractogram of Na-MMT, Zn/MMT/DAO, Mn/MMT/DAO, and Sn/MMT/DAO.

The XRD patterns of Na-MMT and the metal complexes intercalated into MMT (Zn/MMT/DAO, Mn/MMT/DAO, and Sn/MMT/DAO) were recorded at 2θ values between 10° and 90° and are represented in Figure 6. In addition, the prominent absorption peaks and basal spacings (d) of Na-MMT and the intercalated metal complexes are presented in Table 4. Unlike Na-MMT, an additional peak at a 2θ value of approximately 18.20 was observed in the intercalated metal complexes. The reflections of the (001) hkl plane specific to MMT were sharp peaks at 2θ = 7.86° (d = 1.124 nm), 6.04° (d = 1.462 nm), 6.18° (d = 1.429 nm), and 6.16° (d = 1.434 nm) for Na-MMT, Mn/MMT/DAO, Zn/MMT/DAO, and Sn/MMT/DAO, respectively. The interlayer distance (d) of Na-MMT increased from 1.124 nm to 1.429–1.462 nm after intercalation. This was attributed to the loading of the complexes into the interlayer spaces in the structural framework of the MMT. The fundamental peaks of Na-MMT were preserved after the intercalation. These observations indicate that the MMT framework undergoes no significant structural change during intercalation; thus, the crystallinity of MMT is essentially preserved.

### 3.2. Catalytic activity studies

#### 3.2.1. Oxidation of phenol

**Figure 7:**
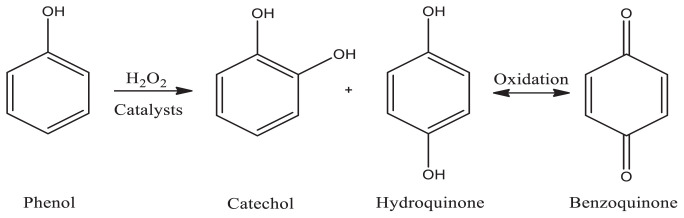
Major oxidation products of phenol.

**Figure 8:**
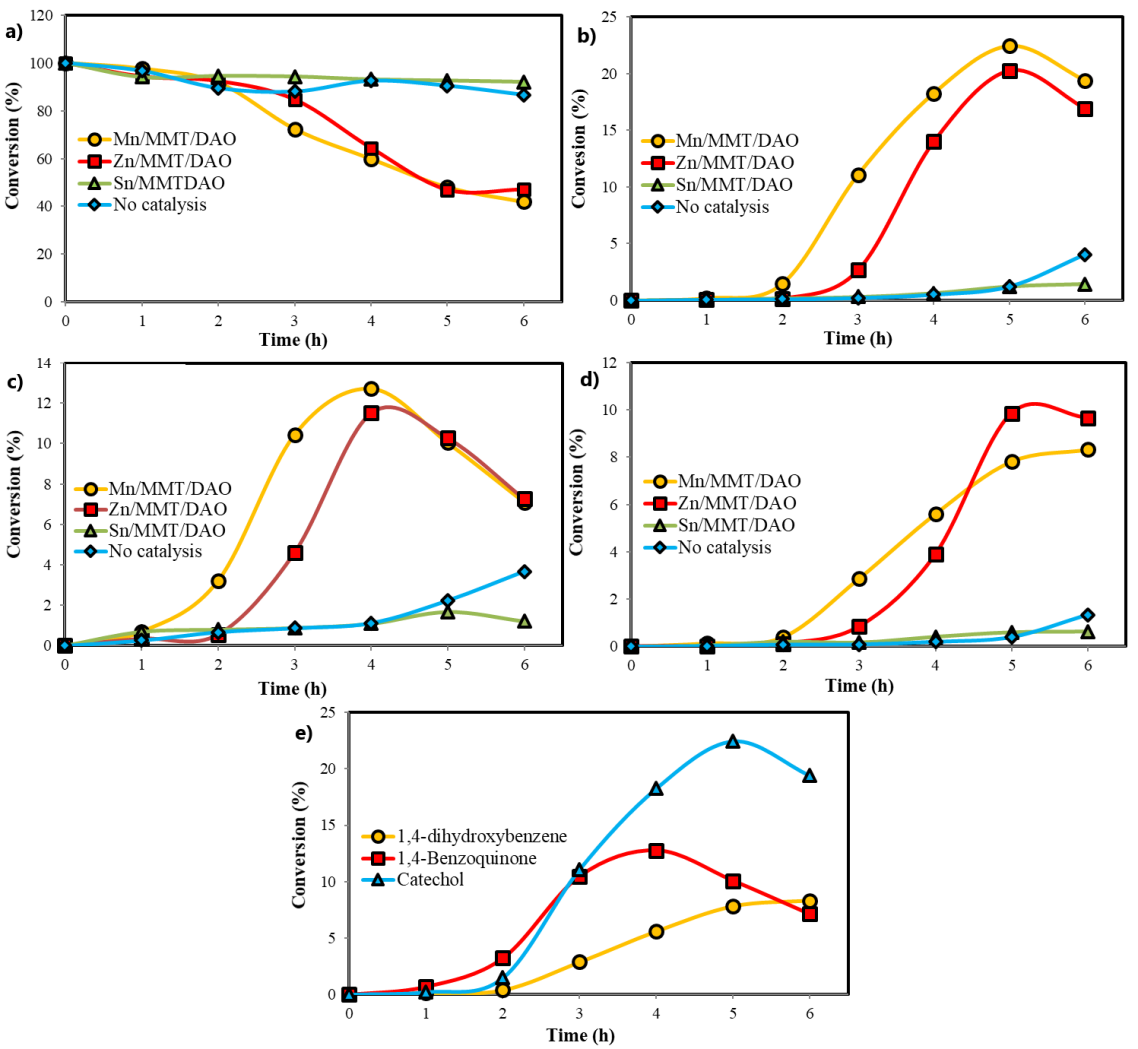
Kinetic plots for a) oxidation of phenol and b) catechol, c) 1,4-benzoquinone, and d) 1,4-dihydroxybenzene formation over different catalysts; e) the three reaction products upon oxidation of phenol over Mn/MMT/DAO.

The catalytic profile of phenol with hydrogen peroxide was studied over metal-oxalamide complexes intercalated into MMT (Mn/MMT/DAO, Zn/MMT/DAO, Sn/MMT/DAO) using acetonitrile as the solvent. The activity of the metal-oxalamide complexes intercalated into MMT was confirmed using a catalyst reaction. It was determined by GC-FID analysis that CAT, BQ, and HQ were the major oxidation products of phenol. The variations and quantities of the reaction products were measured over time and the results are shown in Figure 7. Unlike in the no-catalyst reaction, in the oxidation of phenol with the Sn/MMT/DAO catalyst, all three oxidation products were formed in small amounts after 5 h. While there was no significant change in the conversion rate of phenol over Mn/MMT/DAO and Zn/MMT/DAO catalysts until the first 2 h, the conversion increased after 2 h and three oxidation products were formed at different rates. In addition, as seen in Figures 8a–8e, BQ was formed more predominantly in the first 2 h in the reaction medium, and then CAT and HQ were formed. The maximum reaction yields were 4 h for BQ and 5 h for CAT and HQ. As shown in Table 5, the phenol conversions at 5 h for the Mn/MMT/DAO and Zn/MMT/DAO catalysts were 51.90% and 53.02%, respectively, and the total product yields were 40.32% and 40.40%, respectively. Although the conversion of phenol was not the same, the total product yields were similar. While the CAT yield was higher than that of the Zn/MMT/DAO catalyst at 22.43% for the Mn/MMT/DAO catalyst, the HQ yield was higher than that of the Mn/MMT/DAO catalyst at 9.86% for the Zn/MMT/DAO catalyst.

Additionally, as shown in Table 5, the catalytic activities of materials with only metal intercalated into MMT (Mn/MMT, Zn/MMT, and Sn/MMT) without a ligand (DAO) were examined. The phenol conversions were 59.52% and 60.79% for ligand-free Mn/MMT and Zn/MMT, respectively, and the total yields (three products: CAT, HQ, and BQ) were 26.74% and 29.75%, respectively. Although the conversion of phenol decreased to 51.90% and 53.02%, respectively, for ligand-containing Mn/MMT/DAO and Zn/MMT/DAO, the total yield increased to 40.32% and 40.40%. This shows that metal-oxalamide complexes intercalated into MMT increase the selectivity towards these three products more than those without the ligand, owing to the effect of the ligand. The conversion of phenol was 16.80% for ligand-free Sn/MMT and the total yield was 11.28%. For ligand-containing Sn/MMT/DAO, the conversion of phenol and the total yield decreased to 7.28% and 3.88%, respectively. When the ligand intercalates into Sn/MMT, its catalytic activity decreases. This phenomenon may result from the decrease in the affinity of phenol or its intermediate products for Sn ions as a result of the binding of the ligand to Sn ions.

The effect of the solvent was studied using Mn/MMT/DAO as the representative catalyst. Different solvents including acetonitrile, methanol, 2-propanol, 1,4-dioxane, tetrahydrofuran (THF), *N,N*-dimethylformamide (DMF), and dimethyl sulfoxide (DMSO) were chosen for the oxidation of phenol. As shown in Table 6, the conversion of phenol was 51.90% and the total product yield was 40.32% after 5 h in acetonitrile, which was the best solvent for this reaction.

## 4. Conclusion

The metals and ligands of loadings were examined by EDX/SEM, TG/DTG, FTIR, and elemental analysis and the structural integrity of the clay was verified by XRD throughout the intercalation procedure. The oxidation of phenol with and without the Sn/MMT/DAO catalyst showed poor results. These catalysts catalyze the liquid-phase oxidation of phenol via H_2_O_2_ to catechol as the major product and hydroquinone and benzoquinone as the minor products. Benzoquinone is an oxidation product of hydroquinone. Although the total product yields of the Mn/MMT/DAO and Zn/MMT/DAO catalysts were very similar, the CAT and HQ yields were higher for the Mn/MMT/DAO and Zn/MMT/DAO catalysts, respectively. Mn/MMT/DAO and Zn/MMT/DAO are active catalysts for the oxidation of phenol with H_2_O_2_ compared to the no-catalyst and Sn/MMT/DAO catalyst reactions. It is known that transition metal complexes show higher activity than other metal complexes in the oxidation of organic substances. In parallel with this, it was observed that the pyrazolone-based oxalamide transition metal complexes of Mn and Zn used in this study showed higher catalytic activity than the pyrazolone-based oxalamide metal complexes of Sn. Only the metals were intercalated into MMT and catalytic studies were performed. When the results were compared to those of pyrazolone-based oxalamide metal complexes intercalated into MMT, it was found that the ligand increased the selectivity towards the three products (CAT, HQ, and BQ). These results reflect the important role of the transition metals and ligands used in catalyst preparations for the oxidation of phenol with H_2_O_2_.

## Figures and Tables

**Table 1:** Thermogravimetric analysis data of catalysts.

Material	Temperature range (°C)	Weight loss (%)	Group lost
Na-MMT	20–160	6.60	Free H_2_O
	480–800	4.03	OH group in the clay
Mn/MMT/DAO	20–160	21.59	Free H_2_O
	184–480	8.00	Ligand
	480–800	6.81	OH group in the clay
Zn/MMT/DAO	20–160	7.25	Free H_2_O
	184–480	9.09	Ligand
	480–800	10.44	OH group in the clay
Sn/MMT/DAO	20–160	6.30	Free H_2_O
	184–480	6.21	Ligand
	480–800	4.45	OH group in the clay

**Table 2:** Results of EDX analysis.

Material	Na	K	Mg	Ca	O	P	Si	Ti	Al	Fe	Mn	Zn	Sn	Total
Units	%	%	%	%	%	%	%	%	%	%	%	%	%	%
Na-MMT	2.82	0.34	1.27	1.31	54.36	0.04	26.45	0.32	11.27	1,82	-	-	-	100
Mn/MMT	0.20	0.35	1.39	1.10	59.00	0.08	23.57	0.28	10.32	2.00	1.70	-	-	100
Zn/MMT	-	0.33	1.37	0.62	50.89	0.09	24.00	0.58	9.96	4,25	-	7.91	-	100
Sn/MMT	0.27	-	1.00	1.45	40.87	0.13	16.73	0.45	6.79	3.63	-	-	28.69	100

-: Not detected.

**Table 3:** Results of elemental analysis.

Material	N	C	H	S
Units	%	%	%	%
Na-MMT	-	0.11	3.30	-
Mn/MMT/DAO	1.18	5.47	5.80	-
Zn/MMT/DAO	2.27	7.71	6.31	-
Sn/MMT/DAO	0.91	3.34	4.97	-

-: Not detected.

**Table 4:** Prominent absorption peaks in XRD and basal spacings (d).

Complex	2θ	d (nm)
Na-MMT	7.86, 19.84, 28.18, 35.04	1.124
Mn/MMT/DAO	6.04, 18.16, 19.88, 28.36,35.04	1.462
Zn/MMT/DAO	6.18, 18.18, 19.94, 30.26, 35.04	1.429
Sn/MMT/DAO	6.16, 18.30, 19.96, 26.72, 35.22	1.434

**Table 5:** Effects of different catalysts on oxidation of phenol.

Material	t (h)	Conversion of phenol (%)	Yield (%)			Total yield (%)
			CAT	BQ	HQ	
Mn/MMT/DAO	5	51.90	22.43	10.08	7.81	40.32
Zn/MMT/DAO	5	53.02	20.23	10.31	9.86	40.40
Sn/MMT/DAO	5	7.28	1.21	1.68	0.59	3.48
Mn/MMT	5	59.52	19.08	3.54	4.12	26.74
Zn/MMT	5	60.79	20.99	4.45	4.31	29.75
Sn/MMT	5	16.80	6.36	2.89	2.03	11.28
No catalysis	5	9.47	1.24	2.24	0.40	3.88

**Table 6:** Effects of solvent on oxidation of phenol over Mn/MMT/DAO.

Solvent	t (h)	Conversion of phenol (%)	Yield (%)			Total yield (%)
			CAT	BQ	HQ	
Acetonitrile	5	51.90	22.43	10.08	7.81	40.32
Methanol	5	16.96	9.19	2.26	2.08	13.53
2-Propanol	5	34.92	10.51	0.48	2.13	13.12
1,4-Dioxane	5	36.16	8.21	0.47	1.86	10.54
THF	5	24.83	2.19	0.98	1.31	4.48
DMF	5	18.24	11.54	0.17	5.36	17.07
DMSO	5	4.57	0.01	0.78	0.01	0.80
